# Toward the design of ultrahigh-entropy alloys via mining six million texts

**DOI:** 10.1038/s41467-022-35766-5

**Published:** 2023-01-04

**Authors:** Zongrui Pei, Junqi Yin, Peter K. Liaw, Dierk Raabe

**Affiliations:** 1 New York University, New York, NY 10012 USA; 2 Oak Ridge National Laboratory, Oak Ridge, TN 37831 USA; 3 University of Tennessee, Knoxville, TN 37996 USA; 4 Max-Planck-Institut für Eisenforschung, Düsseldorf, 40237 Germany

**Keywords:** Metals and alloys, Computational methods

## Abstract

It has long been a norm that researchers extract knowledge from literature to design materials. However, the avalanche of publications makes the norm challenging to follow. Text mining (TM) is efficient in extracting information from corpora. Still, it cannot discover materials not present in the corpora, hindering its broader applications in exploring novel materials, such as high-entropy alloys (HEAs). Here we introduce a concept of “context similarity" for selecting chemical elements for HEAs, based on TM models that analyze the abstracts of 6.4 million papers. The method captures the similarity of chemical elements in the context used by scientists. It overcomes the limitations of TM and identifies the Cantor and Senkov HEAs. We demonstrate its screening capability for six- and seven-component lightweight HEAs by finding nearly 500 promising alloys out of 2.6 million candidates. The method thus brings an approach to the development of ultrahigh-entropy alloys and multicomponent materials.

## Abstract (web summary)

The avalanche of publications challenges the norm that researchers extract knowledge from literature to design materials. Here the authors present a text-mining method that is implemented based on the abstracts of 6.4 million papers to enable the design of new high entropy alloys.

## Introduction

Text mining (TM) is an artificial intelligence method to analyze and discover scientific knowledge in literature. It has been used in several fields, such as materials science^1–5^, political science^6,7^, public health^8–11^, etc. TM has the potential for automatic materials discovery given sufficiently large corpora, such as for the material group of high- and medium-entropy alloys (HEAs, MEAs)^12–18^, where more than 10,000 papers have been published^19^. Several TM methods have been suggested that build on corpora as training data^20^. One group of TM algorithms uses vectors to represent words, known as word-embedding algorithms^21–24^. Operations on the vectors provide meaningful information. For example, the difference between vector “FCC" and vector “Al" is approximately equal to that between vector “W" and vector “BCC", since the chemical element “Al" is commonly found with a face-centered-cubic (FCC) crystal structure and “W" with a body-centered-cubic (BCC) structure. These vectors are determined by maximizing the co-occurrence probability of an embedded word and its neighbors within the corpora. The cosine of two vectors measures the similarity of the words they represent. When increasing the frequency of the word “CoCrFeNiV" as the neighbor of “CoCrFeMnNi" by 10 times in a TM (skip-gram) model, its similarity ranking increases by 13 (Supplementary Fig. 1). TM models trained on specially selected corpora are predictive, as the presence of less relevant text items can reduce the relative frequency of keywords^1^.

Here we have developed a highly optimized TM model for metallic materials focusing on HEAs. Unfortunately, TM methods can only identify targeted materials that are in principle already present in the corpora, a fact that does not per se include the discovery of materials. A key challenge in designing HEAs, however, is searching for similar elements with high mutual solubility. To this end, we propose a design concept of “context-similar elements" to overcome this limitation of existing TM methods in this field. The context-similar elements approach aims to capture the similarity of chemical elements in the alloy-design context used by scientists. The similarity in this context is not a metric calculable from simple elemental properties but a more comprehensive one that also reflects researchers’ experience in materials research and design. This approach will enrich the portfolio of existing alloy-design methods and can accelerate the alloy discovery process by replacing the laborious literature search, review, and knowledge extraction with TM models. With this approach researchers with less domain-specific experience can design complex HEAs with many components assisted by TM models that not only “read” huge amounts of publications but also ”analyze” them more context-sensitive.

## Results

**Fig. 1:**
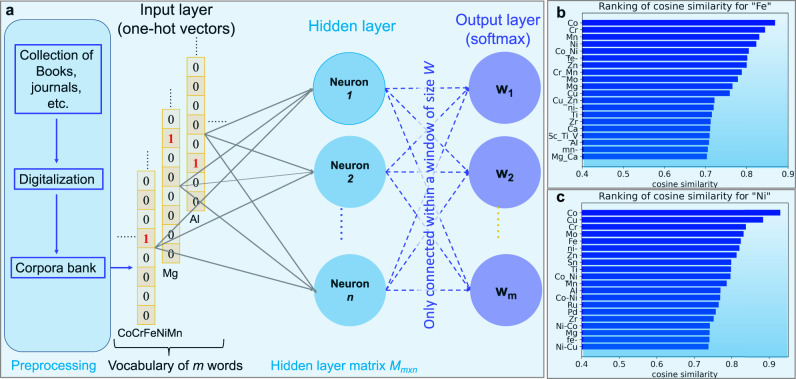
Schematic for the word-embedding model (skip-gram). It has a neural-network structure but with only one hidden layer between the input and output layers^21,22^ (**a**). The training data fed into the model are the processed corpora downloaded from an online database^41^. The corpora are first tokenized into separate words or phrases (combinations of two or more words with unique meanings) and then translated into vectors. In the one-hot representative of a word vector, each word is represented by a sparse vector with only one nonzero element. The word vectors are connected to all neurons in the hidden layer; the latter is also fully connected with the output layer which represents the appearance probabilities of words in their context. For a given window size of the words that define their context, the skip-gram algorithm maximizes the probability of the word that appeared in that context. Once the neural network is optimized, the key information is stored in the hidden layer. As examples of its application, similar words of “Fe" and “Ni" are shown in **b**, **c**, respectively.

Figure 1a shows a schematic for the machine-learning model. We adopt the skip-gram algorithm for our model since the algorithm provides a good compromise between efficiency and accuracy^1,21,22^. It has a neural-network structure with only one hidden layer. Words in the training corpora are firstly encoded into one-hot vectors *w*_*i*_. This means that only one component of each vector assumes a value of “1", which records the word’s location in the whole vocabulary, and the remaining components are “0". These vectors are fed into the neural network as training data. We feed 6.4 million materials-related abstracts plus abstracts on metallic materials into the machine-learning model [see Supplementary Note and Methods]. Here we do not take the weight of the abstracts for metallic materials as a tunable parameter. If we increase its weight, there will not be a convergence trend to test the predictions. Increasing the weight will eventually result in a model without any benefit from other scientific papers. Instead, we tuned different hyperparameters to check the reliability of our models. Constructing a model specifically custom-designed for metallic alloys would be ideally realized by feeding only abstracts of papers that deal with metallic materials. However, papers related to metallic materials represent only a small portion of all scientific papers and thus provide only an insufficient basis for such data-hungry language models. To overcome this problem, our model adopts the commonly used transfer-learning method. We feed all available texts into the skip-gram model, equivalent to constructing a general model, and then feed only texts related to metallic materials, equal to slightly tuning the model for metallic materials. Accurate extraction of the named entities is essential^25^, and particular attention is needed for HEAs. Researchers use different orders of constituents for the same alloy that can be mistaken as different ones. For example, the Cantor alloy, a CoCrFeNiMn compound, can be written in 5! = 120 different name variants by simply rearranging the sequence of the elements, such as CrCoFeMnNi, CoCrFeMnNi, etc. Also, one alloy can be written in various formats like Co-Cr-Fe-Ni-Mn, Co20Cr20Fe20Ni20Mn20, etc. This problem needs particular attention here due to its critical impact on identifying novel massive solid solutions. We alphabetize the chemical elements of one alloy in our skip-gram model [Fig. 1] and knowledge-graph model [Supplementary Fig. 3]. Once a model is trained, the corpora information is encoded into the matrix *M* in the hidden layer. As a simple demonstration of our algorithm, the word vectors *v*_*i*_ for two words “Fe" and “Ni" are used to calculate their respective cosine similarity, i.e., ${S}_{ij}=\cos ({{{{{{\boldsymbol{v}}}}}}}_{i},{{{{{{\boldsymbol{v}}}}}}}_{j})={{{{{{\boldsymbol{v}}}}}}}_{i}\cdot {{{{{{\boldsymbol{v}}}}}}}_{j}/|{{{{{{\boldsymbol{v}}}}}}}_{i}||{{{{{{\boldsymbol{v}}}}}}}_{j}|$. Similar words are ranked and presented in Fig. 1b, c. According to the cosine similarity, the four words that are most similar to Fe are the chemical elements Mn, Co, Ni, and Cr. Likewise, the top four similar words of Ni are Co, Cu, Sn, and Mo. This trend analysis shows that the model can well capture the similarity of words in the context of the chemistry of solid solutions.

### Chemical elements with context similarity

**Fig. 2:**
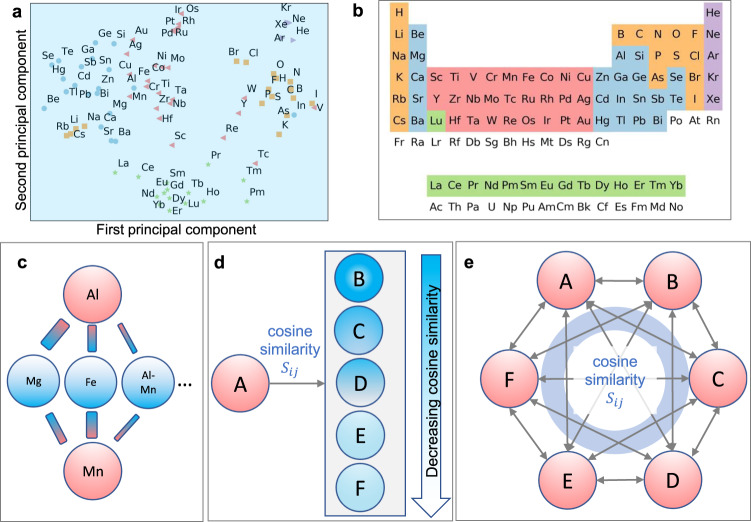
The context-similar elements and their applications to design high-entropy alloys. **a** Chemical elements in the latent space by the principal component analysis (PCA) based on their word vectors. The horizontal and vertical axes are the first two components of PCA. The elements are colored according to the scheme shown in the Periodic Table of Elements (**b**). **c** We explain the reasons for the difference between Mendeleev’s PTE and the chemical elements in the latent space by taking elements Al and Mn as examples. The thicknesses of the sticks represent the connection strengths of words (similarity). We propose two methods to design high-entropy alloys. These methods are illustrated in **d**, **e**, taking six-component alloys as an example. The first method starts with one element that must be included and select its four most similar elements according to the cosine similarity. The second method considers all participating elements equally. The cosine similarity *S*_*ij*_ of any two elements are averaged to measure its potency as a candidate.

The distribution of elements is visualized in Fig. 2a, using the color map defined in Fig. 2b. Elements with similar chemical features are grouped together. The context-similar version of the Periodic Table of the Elements (PTE) includes rich information about how they were used in the enormous amount of literature. Suppose two chemical elements appear in a similar context. They are close in the vector space but not necessarily in Mendeleev’s PTE. For example, Al is not the neighbor of Mn and Cr in Mendeleev’s PTE. However, they are neighbors in the latent space of our word-embedding model. Mendeleev’s PTE has its physical origin in quantum mechanics, while our machine-learning model is based on the appearance of the elements in various research contexts. We take Al and Mn as an example pair to show how they can be grouped in the two representation forms (in the latent space of our machine-learning model and Mendeleev’s PTE) [Fig. 2c]. Elements Al and Mn share many similar neighboring words, such as Fe and Mg, since they are often used as solute atoms in steels and magnesium alloys, albeit with different prevalence. This fact increases the co-occurrence probability that puts them close in the latent space. Therefore, the context similarity of elements reflects better how researchers used them to synthesize actual materials, which is precisely the experience needed to design alloys.

### Design of alloys based on context similarity

Exploration of the vast space for HEA design challenges traditional alloy-design strategies^19^ and requires an intelligent choice of elements, particularly when targeting solid solutions. It is important to overcome this challenge, driven by the need for high-performance materials, for which HEAs with multiple components are promising candidates. In this regard, the concept of publication-based context-similar elements provides unexplored opportunities. We propose two different methods to use word vectors in the HEA design [see Fig. 2d, e]. One is to start with a specific element A, which is for some reason preferred to be included as a chemical component, and identify the most similar elements according to the cosine similarity {S_*ij*_}. For example, for a Fe-including quaternary HEA, the top three candidates are Mn, Co, and Ni according to S_*ij*_ [Fig. 1b]. Instead, if our target is a HEA with Ni, the top three candidates are Co, Cu, and Sn. Sn is not close to Ni in the PTE but often appears in Ni-Sn alloys, reflecting the difference between our text similarity and the PTE again. In a second method, we first select *M* promising elements {*E*_*i*_}${}_{i=1}^{M}$ and consider their cosine similarity equally [Fig. 2e]. For each *N*-component HEA, we average over the similarity of each element pair *S*_*ij*_, i.e.,

1 $$\bar{S}=\frac{1}{N(N-1)/2}\mathop{\sum }\limits_{i=0}^{N-1}\mathop{\sum }\limits_{j=i+1}^{N-1}\cos ({{{{{{\boldsymbol{v}}}}}}}_{i},{{{{{{\boldsymbol{v}}}}}}}_{j}).$$

Here ***v***_*i*_ and ***v***_*j*_ are the word vectors of elements *i* and *j* in the *N*-component HEA. We rank the alloy candidates according to $\bar{S}$ and pick the top candidates as the most promising ones. This method treats all elements equally and thus is consistent with the alloy-design spirit of multi-principal HEAs.

### Body-centered-cubic high-entropy alloys

**Fig. 3:**
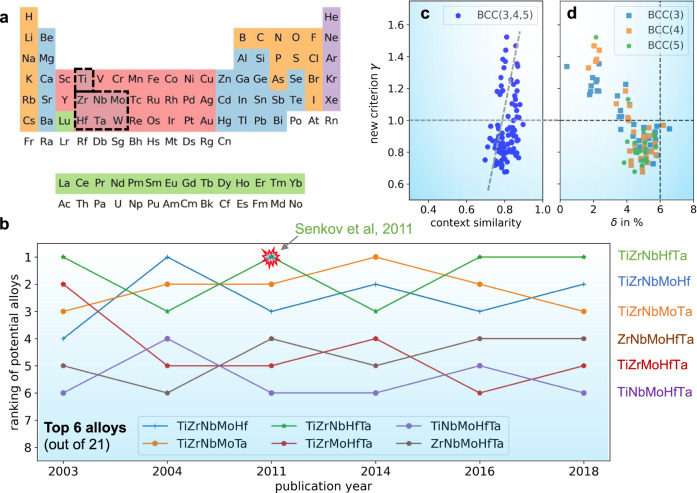
The context-similar elements and their applications for BCC high-entropy alloys. **a** Taking the transition elements (Ti, Zr, Nb, Mo, Hf, Ta, and W) as an example, we show that the tendency to form solid solutions is correlated to the newly defined context similarity. For better visualization, we only consider here three-, four-, and five-component equiatomic alloys. **b** The five-component alloys are ranked by their context similarity for different publication years. The top three alloys include the Senkov alloy of TiZrNbHfTa, TiZrNbMoHf, and TiZrMoHfTa. The Senkov alloy was proposed in 2011 by Senkov et al.^48^. Other promising ones include ZrNbMoHfTa, TiNbMoHfTa, TiZrNbMoTa. **c** The context similarity $\bar{S}$ is strongly correlated with the thermodynamics-based *γ* parameter. **d** More details on the *γ* parameter when using it along with the lattice distortion *δ* to predict the solid solutions.

Exploration of refractory BCC HEAs with beneficial properties attracts high attention currently^26–29^. We demonstrate the approach of the “context similar" elements in Fig. 3 by limiting our candidate elements to common transition-metal elements. As an example and to demonstrate the predictive strength of our method, the newly defined $\bar{S}$ parameter in Eq. (1) is adopted to design five-component HEAs. We focus on seven elements, which yield 21 combinations (alloys). Ti, Zr, Nb, Mo, Hf, Ta, and W are among the most common transition metals, and some of them appear in the Senkov alloy TiZrNbHfTa^28^. In order to critically test the predictive power of our approach and understand the trend of HEAs, we train individual models for the representative years. Each model adopts training data of the publications that appeared only in its corresponding year. The only exception is the model of 2003 that adopts all abstracts up to that year. The idea behind that is to test if our modified TM model could have predicted an alloy with only the knowledge that was available up to the year 2003 (i.e., the Senkov alloy had not yet been discovered, but it was found only 8 years later). The results predicted by the models are shown in Fig. 3b. Among the 21 candidates, the Senkov alloy is continually ranked among the top three materials. In 2011 when the alloy was finally discovered and synthesized, and more recently 2016 and 2018, the Senkov alloy is at the absolute top of the list. This means that our context-sensitive TM model would have suggested this specific HEA at least eight years before conventional alloy-design approaches found it.

In order to explore if any physical meaning is connected to our $\bar{S}$ parameter, we adopt our simple, approximate method to predict solid solutions and calculate the so-called *γ* parameter^30^. Simply put, the parameter is a ratio between the approximate Gibbs free energy (usually a negative number) of the HEA *G*_*N*_ and that of its binary subsystems *G*_2_. If an alloy has a *γ*-value larger than 1, it is likely to be a solid solution rather than a multi-phase alloy. Furthermore, the parameter is found to be linearly and positively correlated with $\bar{S}$ [Fig. 3c]. This feature indicates that a larger value of $\bar{S}$ offers more promising candidates as solid solutions. The prediction of solid solutions using the *γ* parameter can be improved with the lattice-misfit parameter *δ* as an additional physics-based descriptor^30^, which measures the lattice distortion due to the different atomic sizes [see Fig. 3d]. The use of these two additional descriptors reveals that only a tiny fraction of the ternary, quaternary, and quinternary alloys are solid solutions with the highest similarity scores. The majority of them are multi-phase alloys.

### Face-centered-cubic high-entropy alloys

Similar to the BCC HEAs, the averaged context similarity $\bar{S}$ is calculated for a group of FCC HEAs and shown in Supplementary Figure 2. Again, we limit the constitutional elements to the transition-metal elements from V to Cu of the third group. Taking the five-component alloys as an example, we show the similarity $\bar{S}$ for individual years in Supplementary Fig. 2a. This test protocol shows that the concept effectively identifies HEAs long before they were found by conventional alloy-design methods. The Cantor alloy was first reported in 2004, but it was ranked as the second most promising solid-solution HEA by our method already before 2004. The seminal paper of Cantor et al. did not receive much attention immediately after its publication, but its impact has increased exponentially since the last decade^19^. This trend is correctly reflected by its ranking in Supplementary Fig. 2a. The second and third most promising HEAs are MnFeCoNiCu^31^ and CrFeCoNiCu^13^, which were also synthesized. We also calculate their tendency to form solid solutions by using the *γ* parameter^30^. As presented in Supplementary Fig. 2b, the two quantities are linearly correlated, similar to the case of the BCC HEAs. This trend further confirms the significance of the $\bar{S}$ parameter in screening for high-entropy solid solutions.

### Combination with Integrated Computational Materials Engineering (ICME) methods to design ultrahigh-entropy alloys

The method of “context similarity" picks element candidates for HEAs, which is the first step for designing high-entropy solid solutions. Then, various procedures can be developed for further screening, refining, and filtering the results, assisted by the methods grouped under the umbrella of ICME (integrated computational materials engineering)^32,33^ and included in the materials genome initiative^34^. ICME is an approach for designing materials and microstructures using mean-field thermodynamics and kinetics tools as well as ab-initio and structure-property simulation methods. A few examples are provided below to show how to integrate the context-similarity method with ICME to accelerate the design process.

In the first example, we screen for alloys based on their mechanical properties. Critical mechanical properties include for instance creep, ductility, and yield stress. Here we focus on one important mechanism behind these features, i.e., solid-solution strengthening *σ*_*y*_, as it provides an essential contribution to the yield stress^35,36^. We adopt a model developed recently by Varvenne et al.^35^. The full details of the model are available in the reference and supplementary material. Supplementary Fig. 2d shows the solid solution strengthening predicted at 300 K for the top six FCC alloy candidates. The CrFeCoNiCu alloy has the largest strengthening effect *σ*_*y*_ of ~290 MPa, followed by CrMnFeCoCu with a comparable value. The strengthening effect of about 146 MPa for the Cantor alloy is fairly consistent with the experimental measurement of 125 MPa^35,37^. More validations of the methods can be found elsewhere^35,38^. These results show that alloys with optimal mechanical properties can be designed jointly with the TM-based $\bar{S}$ parameter.

**Fig. 4:**
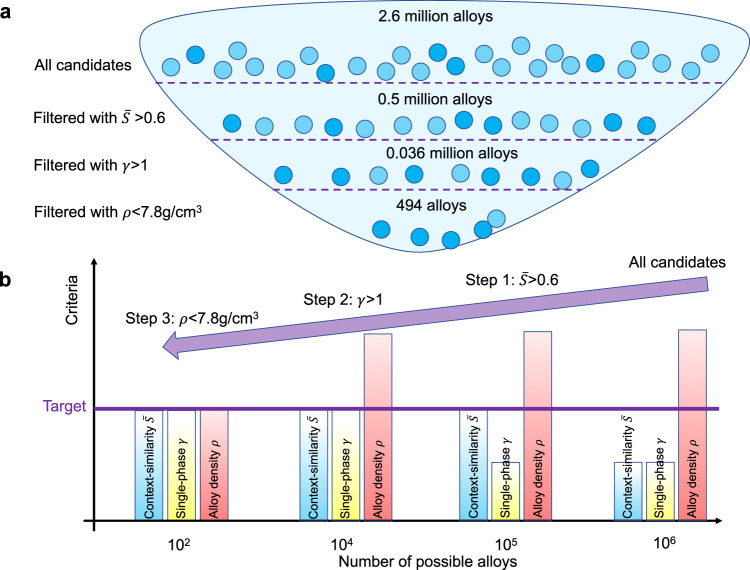
An exemplary design of lightweight high-entropy alloys with more than five components. **a** We show the example study conducted for six- and seven-component single-phase, equiatomic alloys, limited to 30 transition-metal elements. The first step is to calculate the average context similarity $\bar{S}$ and select alloys with a $\bar{S} \, > \, 0.6$. The distributions of $\bar{S}$ for all these candidates are shown in Supplementary Fig. 5. The second filtering step is to find in this subset those alloys that are likely in the form of solid solutions assisted by the thermodynamics-based rule *γ* > 1^30^. The third step is to select the alloys with a density smaller than iron, i.e., *ρ* < 7.8 g/cm^3^. Here we picked all alloys with a mass density below that of iron. **b** Along this filtering workflow, the number of possible alloys decreases from 10^6^ to 10^2^. Alloys in the shortlist are candidate materials for synthesis and testing.

In the second example, we aim to use our method and go beyond the established high entropy systems and design six- and seven-component, lightweight, single-phase equiatomic HEAs. This example has been partly motivated by Cantor, who calls for a bolder design of HEAs, also considering materials beyond five components^19^. To tackle this challenge, we design a workflow comprised of multiple steps and elaborate on the application and its statistical aspects in Fig. 4a, b. The screening consists of three steps with three adjustable criteria, i.e., (i) context similarity $\bar{S} \, > \, 0.6$; (ii) thermodynamics-based solid solution parameter *γ* > 1, and (iii) the alloy’s mass density *ρ* < 7.8 g/cm^3^ (density of iron). In the current example, these three criteria have been selected for the sake of demonstration. The high-throughput screening has been limited to 30 transition-metal elements [see supplementary text for the list]. The total number of alloy candidates before screening has been 2.6 million. After each step, we excluded sets with 1–2 orders of magnitude of alloys. Eventually, only 494 HEAs remained and are promising for synthesis in experiments.

More specifically, the top-ranked alloys along these three categories are TiCrFeCoNiMo (six-component), TiCrFeCoNiCuZn (seven-component), and TiFeCoNiCuZn (six-component) according to the context similarity $\bar{S}$; six-component VCrMnFeCoNi, VCrMnFeCoCu, and VCrMnFeNiCu following the thermodynamics-based parameter *γ*; and six-component ScTiZnZrAgCd, TiVCrMnFeZn, and TiVCrMnCuZn regarding the lowest mass density. These results demonstrate concrete design suggestions identified directly and autonomously via our TM modeling approach from the existing wealth of published literature. The list of the 494 identified alloys can be found in the supplementary data.

### Searching for existing HEAs to avoid redesigning alloys

One of the crucial tasks for materials designers is to check if the targeted alloys have been already proposed and synthesized before. This task becomes increasingly important in the ever-growing information avalanche. When studying HEAs, an additional challenge arises from their non-standardized naming system. Therefore, there is an urgent need to build TM models in which all HEAs are standardized with unique names, as in chemistry. It would then be much easier to check if an alloy has already been synthesized before or not. The knowledge graph (KG) approach has demonstrated its usefulness in quickly retrieving the required information^2^. KG is a graph-structured data model that links entities such as alloys and their properties through various relations (i.e., edge words) [see Supplementary Fig. 3a]. The Google knowledge graph is for general-purpose applications, yet, it is not specialized for extracting useful information from research corpora^39^. So, for example, it cannot tell if CoCrFeMnNi and NiMnFeCrCo are the same materials or not. Here we propose a KG for alloys, focusing on HEAs, i.e., an alloyKG, as an acronym for the knowledge graph for alloys. Their constituent elements are ordered alphabetically according to their symbols. We connect the HEAs with the DOI’s (Digital Object Identifiers) of the papers in which they appeared. Essential authors in the field and their specific contributions can also be identified for further processing. In Supplementary Fig. 3b, we show the results for an exemplary search using our alloyKG approach with the keyword “CoCrFeMnNi" and the edge phrase “mentioned by". Since the naming system is standardized in alloyKG, every arrangement of the 120 possibilities for a five-component alloy gives the same results. The retrieval yields the papers (represented by their DOI’s) that mentioned the alloys.

## Discussion

One of the most important directions for the HEA community is to explore the vast compositional space of HEAs with more components, such as six or seven components, and not limit the search to five or fewer components. Irrespective of the success of traditional TM methods, one of their principal shortcomings is that they cannot readily design alloys that do not appear in the corpora. This challenge has been overcome by our current “context similarity element” concept. One unique and characteristic feature of HEAs is that they are (ideally) solid solutions, which means we can screen for similar element candidates and ignore their specific concentrations first. This strategy is one of the main reasons why the concept works specifically well for the current alloy-design task. The transferability of our method has been carefully tested for a wide range of alloys from medium, high, and even ultrahigh-entropy alloys, with both BCC and FCC crystal structures. The method is applicable to the design of different HEAs, and there is no specific additional need to fine-tune the model. Another benefit of the approach is that the selected alloy groups can be further refined, after pre-screening promising composition spaces by our TM approach, by using further filtering criteria from the established ICME toolbox„ such as thermodynamic, kinetic, structural, and/or property databases and simulations. This hybrid alloy-design concept, combining TM-based pre-screening of the practically infinite chemical composition space and subsequent physics-based filtering, paves a pathway towards a closed-loop materials design approach that is characterized by the following specific steps: (i) fully automatically reading and autonomously analyzing millions of papers, (ii) searching for a specific set of chemical elements and suited alloy ingredients, (iii) proposing alloy candidates, (iv) calculating properties of the alloys, (iv) selecting alloys based on the targeted properties, and (v) identifying and excluding alloys that were already synthesized and casting all results into a recommendation list. With this approach, even inexperienced users with less domain knowledge in the field of alloy design can develop complex materials with many components assisted by TM models and a huge body of scientific publication corpora.

The word vectors of the chemical elements can reflect the rise of specific HEAs, quantified by changes in the cosine similarity *S*_*ij*_ [Supplementary Fig. 4]. The Cantor alloy of CoCrFeMnNi was proposed in 2004^14^. Prior to that year, the most similar elements to Fe, according to our similarity index, are Cr, Mn, Mg, and Al. Given the increasing relevance of the Cantor alloy and its subsystems, the top four most similar elements to Fe, when extracted from context mining, are only the elements in the Cantor alloy, at least since 2014. In 2014, several milestone papers appeared about the Cantor alloy. For example, it was found to have good ductility and toughness even at cryogenic temperatures^40^. In a different study of the same year, several compositional subsystems of the Cantor alloy were explored^37^. Stable FCC systems were identified, including the equiatomic CrCoNi solid solution. These investigations and many others pushed Mn, Co, Ni, and Cr into the top similarity list for Fe. We trained different models to test the stability of our method. When the window size is changed from 8 to 10, and the dimension (number of neurons in the hidden layer) from 200 to 300, the most similar words of “alloy_HEA" remain almost the same [see Supplementary Fig. 6], but the training time increases significantly. We also applied the models to calculate the context similarity, taking BCC HEAs as an example [see Supplementary Fig. 7]. The representative Senkov alloy TiZrNbHfTa is ranked number one by these models. All these HEAs follow the same order except for the TiZrNbMoHf and TiZrNbMoTa to switch their positions. This trend again shows a model with 200-dimensional word vectors and a window size of 8 is sufficient for designing alloys.

In summary, we proposed a concept for the systematic and automatic search for “context similarity elements” and demonstrated its successful application for the design of high-component high-entropy alloys. The method overcomes the common problem of traditional text mining methods that can only explore existing materials and enables us to design alloys that do not appear in the training corpora. As a demonstration, we show that the approach would have successfully identified the representative FCC Cantor and BCC Senkov alloys as the most promising high-entropy alloys, long before they had been actually discovered and synthesized. We also find that the context similarity is strongly correlated to a thermodynamics-based rule proposed by us in a previous study^30^. This trend indicates that this thermodynamic alloy-design parameter adequately captures the tendency of solid solution formation. We also show that this method can be integrated with other ICME methods deemed vital for the materials genome initiative. Furthermore, we designed a workflow for high-throughput screening of lightweight six- and seven-component HEAs. We show that the method has the potential to realize the ambitious aim to find high-component HEAs, as recently proposed by Cantor^19^. It also equips the research community with a general tool for the efficient discovery of materials.

## Methods

### Data collection and processing

Scientific texts appear in various formats, such as books, journals, etc., either in printed or electronic versions. The first step for corpora collection is to unify all these texts in a single digital format that can be directly used in machine-learning models (Fig. 1a of the main text). Here the training corpora of 6.4 million abstracts are downloaded through the ELSEVIER Scopus API^41^. The latter can retrieve abstracts in bulk with the journal ISSN and publishing year as input. We use the ISSN list generated by Tshitoyan et al.^1^ as the starting point. The abstracts are stored in JSON format along with the metadata, such as authors, years of publication, keywords, journals, etc. In addition, we also manually add important journals and abstracts for HEAs that are absent in the first round of abstract collections. The representative journals for metallic materials of the past two decades include Acta Mater., Journal of Alloys and Compound, Materials Science and Engineering: A, and Advanced Engineering Materials. Note that there is a weekly download quota for regular Scopus developer API. The entire collection process of 6.4 million abstracts can take several months.

### Machine-learning model

The skip-gram algorithm is adopted in this study. It has a neural-network structure but only with one hidden layer. Words in the training corpora are firstly encoded into one-hot vectors *w*_*i*_. One component of each vector is “1", which records the word’s location in the whole vocabulary, and the rest are zeros. These vectors are fed into the neural network as the training data. The training objective is to maximize the probability of each word in their context defined by a window size *n* (8), which is also the cost function to be optimized. The left and right eight words are considered the neighbors of the word in one basic unit, i.e., one abstract. The skip-gram model is trained for 30 epochs, and more epochs do not significantly improve the model performance. In the trained model, the key information of the corpora is encoded into the matrix *M* in the hidden layer. Multiplying *w*_*i*_ by *M* we obtain a representative vector *v*_*i*_ of 200 dimensions for the word *i*. There is no need to revisit the neural network for applications of the model. Words that are semantically or grammatically similar correspond to vectors that can reflect the similarity. The vector *v*_*i*_ has many interesting properties, such as compositionality and cosine similarity, as mentioned previously.

Here we feed the 6.4 million abstracts plus abstracts on metallic materials into the machine-learning model. The list of journals whose abstracts are duplicated is described in the supplementary material. The consequence is we place a double weight on the metallic materials. As a result, the specially tailored model is expected to work better for metallic materials than previous models.

### Thermodynamic rule

Our thermodynamics-based rule was initially derived and published in ref. 30. It provides a systematic method to calculate the free energies Δ*G*_*N*_ for a given N-component system and Δ*G*_2_ for all its binaries. If Δ*G*_*N*_ is the lowest, the multicomponent system is a single-phase alloy; otherwise, it is a multi-phase alloy. For convenience, we define a parameter *γ* to describe this criterion, i.e.,

2 $$\gamma :=\left\{\begin{array}{ll}{{\Delta }}{G}_{N}/\min ({{\Delta }}{G}_{2}) &\,{{\mbox{if}}}\,\min ({{\Delta }}{G}_{2}) \, < \, 0; \hfill \\ -{{\Delta }}{G}_{N}/\min ({{\Delta }}{G}_{2}) &\,{{\mbox{if}}}\,{{\Delta }}{G}_{N} \, < \, 0\,{{\mbox{and}}}\,\min ({{\Delta }}{G}_{2}) \, > \, 0.\end{array}\right.$$

The criterion now becomes *γ* ≥ 1.

### Density functional theory calculations

Density functional theory^42,43^ simulations are carried out using Vienna Ab-initio Simulation Package (version 5.4.4)^44^ to obtain the optimal volumes in a designated crystal structure (here FCC). The generalized gradient approximation parametrized by Perdew-Burke-Ernzerhof^45^ is used to calculate the electronic exchange-correlation interaction, and the Kohn–Sham equation is solved using the projector augmented wave method^46^, where the Brillouin zone is sampled using Monkhorst-Pack scheme^47^. The atomic configurations of elements in the pseudopotentials used in our calculations are V [Ne3s^2^]3p^6^3d^3^4s^2^, Cr [Ar]3d^5^4s^1^, Mn [Ar]3d^6^4s^1^, Fe [Ar]3d^7^4s^1^, Co [Ar]3d^8^4s^1^, Ni [Ar]3d^8^4s^2^, and Cu [Ar]3d^9^4s^1^. The relaxation stops when the energy difference between ionic steps is smaller than 10^−5^ eV. A plane wave cutoff of 400 eV and the k-point meshes of 10 × 10 × 10 for the Brillouin zone are used. A supercell size of four atoms is adopted for pure elements and FCC structure in this study. In these calculations, only volume relaxation is needed.

## Supplementary information

Supplementary Information

Description of Supplementary Data 1

Supplementary Data 1

## Data Availability

The article DOIs used to generate the training corpora in this study have been deposited in our GitHub repository under the accession link (https://github.com/peizong/alloy2vec). The raw training corpora data are protected and not shared due to the data privacy rules of Elsevier. Users can download it after they open an Elsevier account since all the papers are stored in their database. Details and guidelines to use the API and papers provided by Elsevier are here: https://dev.elsevier.com. Any reader can register there and receive an API account to reproduce the results. All copyright rules explained by Elsevier on that webpage must be followed.

## Code availability

The *alloy2vec* code is available in GitHub (see Data Availability), and can also be downloaded through 10.5281/zenodo.7337034.
