# Author Correction: Toward the design of ultrahigh-entropy alloys via mining six million texts

**DOI:** 10.1038/s41467-023-39406-4

**Published:** 2023-06-16

**Authors:** Zongrui Pei, Junqi Yin, Peter K. Liaw, Dierk Raabe

**Affiliations:** 1 New York University, New York, NY 10012 USA; 2 Oak Ridge National Laboratory, Oak Ridge, TN 37831 USA; 3 University of Tennessee, Knoxville, TN 37996 USA; 4 Max-Planck-Institut für Eisenforschung, Düsseldorf, 40237 Germany

**Keywords:** Metals and alloys, Computational methods

Correction to: *Nature Communications* 10.1038/s41467-022-35766-5, published online 04 January 2023

The HTML version of this Article incorrectly omits Supplementary Data. Supplementary Data can be found as Supplementary Information associate with the original article and this Correction, and the description can be found in the file entitled “Description of Additional Supplementary Files”.

## Supplementary information

Description of Additional Supplementary Files

Supplementary Data 1

