# Supplementary material for: Toward the design of ultrahigh-entropy alloys via mining six million texts
Source: Nat Commun. 2023 Jan 4;14:54. doi: 10.1038/s41467-022-35766-5 (PMC9813346; doi:10.1038/s41467-022-35766-5)
Supplement: Supplementary file 1 — Supplementary Information [file 41467_2022_35766_MOESM1_ESM.pdf]

Supplementary Materials for Toward the design of ultrahigh-entropy alloys via mining six million texts

Zongrui Pei,[1,2*†] Junqi Yin[2†], Peter K. Liaw[3], Dierk Raabe[4]

[1]New York University, New York, NY10012, USA
[2]Oak Ridge National Laboratory, Oak Ridge, TN37831, USA
[3] The University of Tennessee, Knoxville, TN 37996, USA
[4]Max-Planck-Institut für Eisenforschung, Düsseldorf, 40237, Germany
[†]Equal contribution.

Supplementary Note

We provide five figures supplementary to the main text. Figure 1 shows the sensitivity of the word ranking according to cosine similarity. By increasing the frequency of the word ”CoCrFeNiV” by 10 times, its ranking in the top list of ”CoCrFeMnNi” changes by 13 (from 30th to 17th). Figure 2 shows the text-mining-based technique that can be used to identify alloys ahead of their discovery. More importantly, our new method can pick new materials with many components, paving the way to the design of ultrahigh-entropy alloys. Figure 4 shows the top list of word ”Fe” as a function of publication time. Figure 3 shows the knowledge-graph (KG) model and its applications to retrieve the representative BCC and FCC high-entropy alloys. Figure 5 shows the distributions of the similarity quantity defined in this study. The distributions both for the six- and seven-component HEAs are demonstrated.

List of double weighted journals

Abstracts that appeared in the following journals are duplicated have a double weight than other journals. The journals are well known to focus on metallic alloys, including high-entropy alloys.

”Acta Materialia”, ”Scripta Materialia”, ”Materials and Design”, ”Journal of Alloys and Compounds”, ”Materials Science and Engineering A: Structural Materials: Properties, Microstructure and Processing”, ”Intermetallics”, ”Metallurgical and Materials Transactions A”, ”Annual Review of Materials Research”, ”Progress in Materials Science”, ”Science”, ”Nature”, ”Nature Reviews Materials”,”Physical Review Materials”, ”Advanced Materials”, ”Advanced Engineering Materials”, ”International Journal of Plasticity”, ”Current Opinion in Solid State and Materials Science”, ”Journal of Materials Science and Technology”

List of the 30 transition metal elements for the lightweight-HEA screening

Sc, Ti, V, Cr, Mn, Fe, Co, Ni, Cu, Zn, Y, Zr, Nb, Mo, Tc, Ru, Rh, Pd, Ag, Cd, La, Hf, Ta, W, Re, Os, Ir, Pt, Au, Hg

The yield stresses at 0K, 300K and 600K

We used the Varvenne model [1] to calculate the yield stresses for the FCC HEAs. Full details are available in the reference. We describe only the formula to calculate τ at 0K, i.e., $\sigma_y = 0.051 M \alpha^{-\frac{1}{3}} K_\tau f_\tau \left[\sum_n c_n \Delta V_n^2 \right]^{\frac{2}{3}}$. Here M is the Taylor factor of 3.06, the line tension constant $\alpha = 0.123$ and the geometric constant for a wide-core dislocation $f_\tau = 0.35$ and the elastic constant K_τ. The critical parameter is the total volume misfit $\sum_n c_n \Delta V_n^2$, calculated using density functional theory. The results are listed in Supplementary Table 1.

Extended data figures

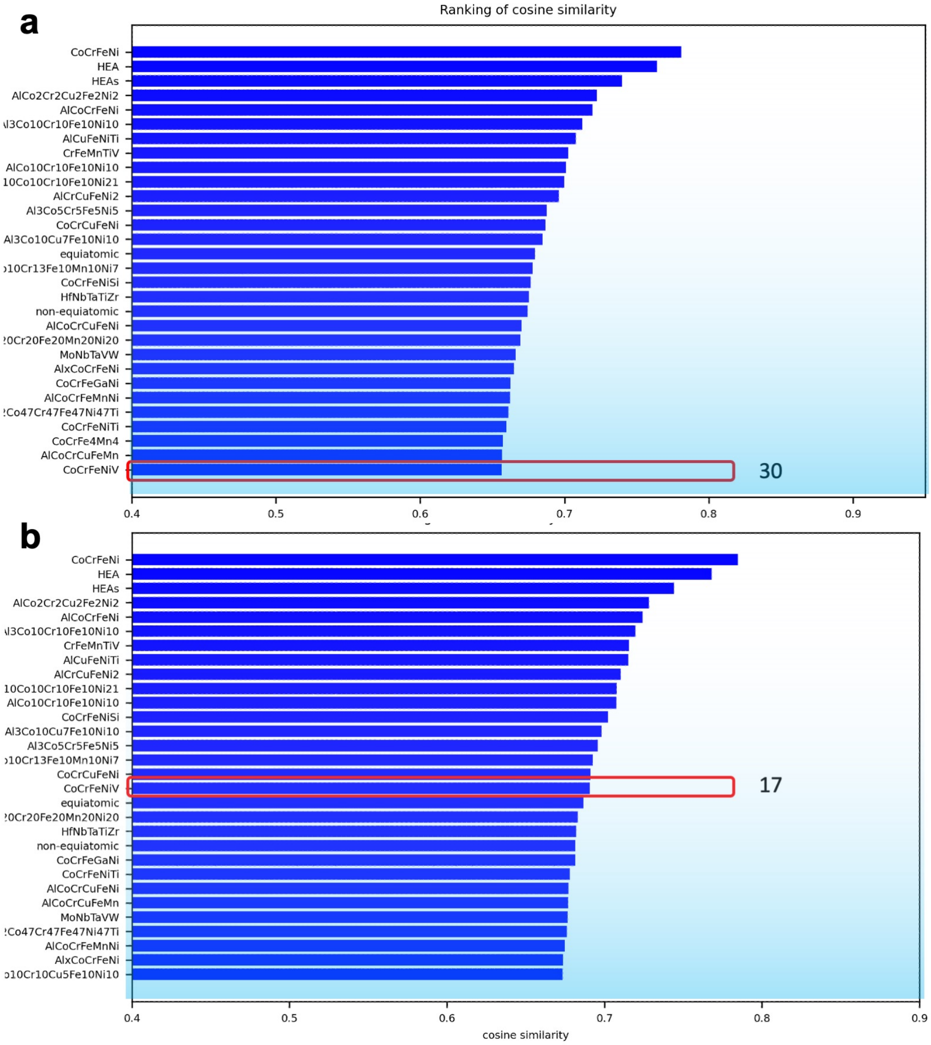

Supplementary Figure 1: The ranking of similar words for "CoCrFeMnNi". **a**, The list calculated from the skip-gram model without the duplicated abstracts of metallic materials; **b**, the same model is adopted and retrained with a new piece of text. In the new text, the appearance of "CoCrFeNiV" is increased by ten times as the neighboring word of "CoCrFeMnNi".

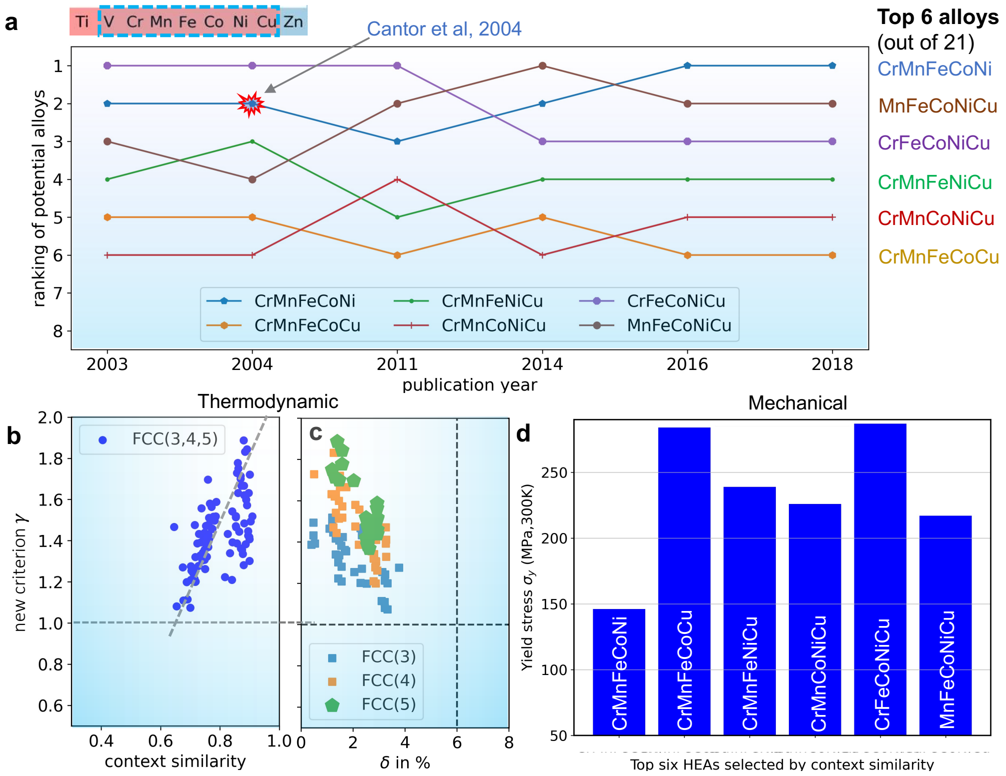

Supplementary Figure 2: Integrating the new TM-based technique with other ICME methods for alloy design. **a**, Taking the transition elements (from V to Cu) as an example, the five-component alloys are ranked by their context similarities for different publication years. Even before 2004, when it was proposed [2], the Cantor alloy is highly ranked as the second most promising solid solution and later assumes even the first position due to the increasing number of research works about this material and its subsystems. The other three materials had also been synthesized by the HEA community. Other promising materials were also synthesized, such as CrMnFeNiCu and CoCrFeNiCu in 2016 [3], and CrMnFeCoCu, CrMnCoNiCu and FeCoNiCuV in 2008 [4]. **b**, The capabilities of context similarity to screen for promising solid solutions. We show that the tendency to form solid solutions is correlated to the context similarity $\bar{S}$. In **c**, more details on the application of the similarity parameter γ and the lattice distortion δ are provided. **d**, The predicted yield stress at room temperature is used as a descriptor to screen for suited alloys. As examples, only the top six FCC HEAs selected by $\bar{S}$ are shown.

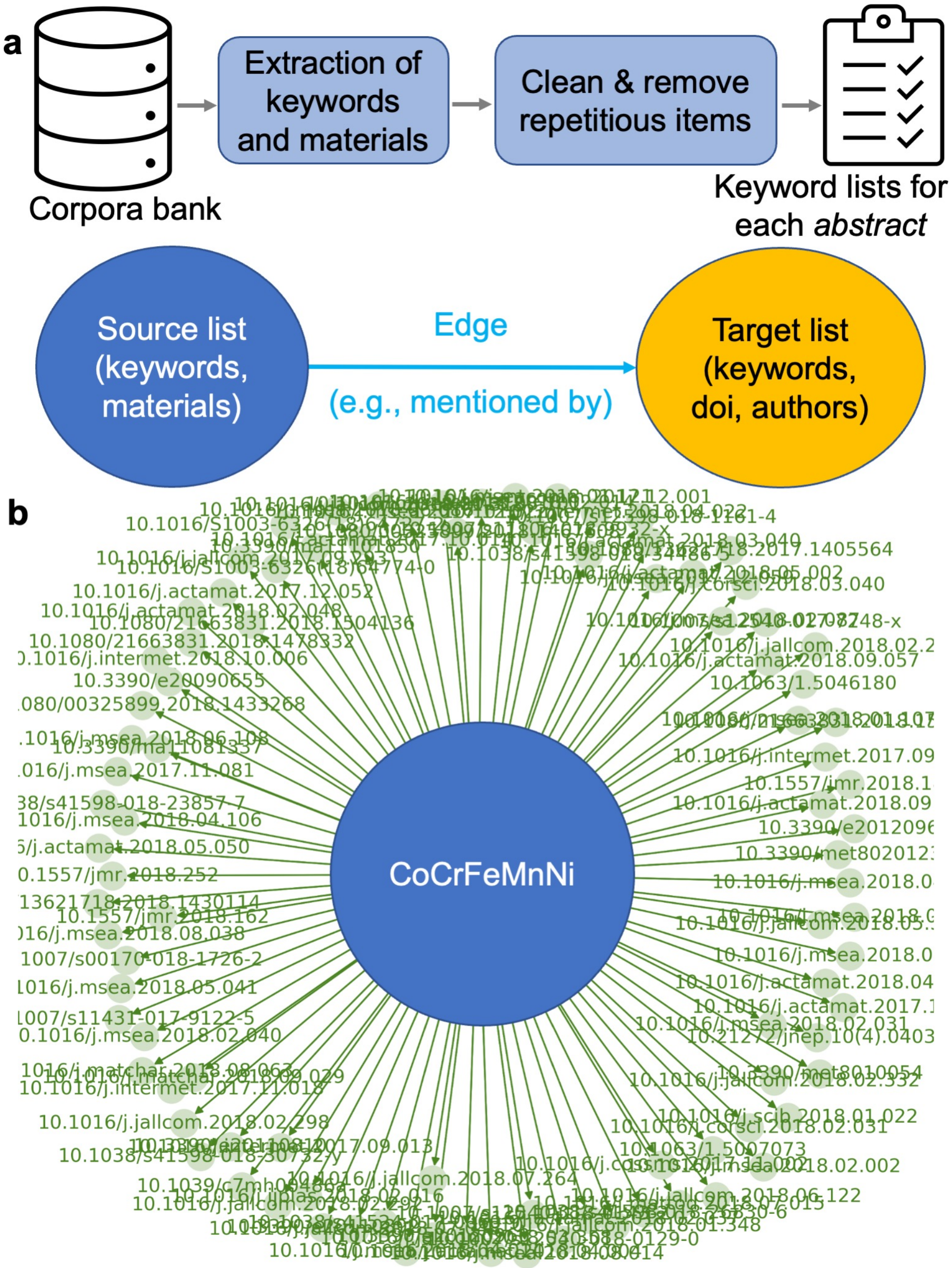

Supplementary Figure 3: Knowledge graph for high-entropy alloys. **a**, The key steps to build a knowledge-graph model and the model structure. **b**, Searching alloyKG using keyword “CoCr-FeMnNi” (Cantor alloy).

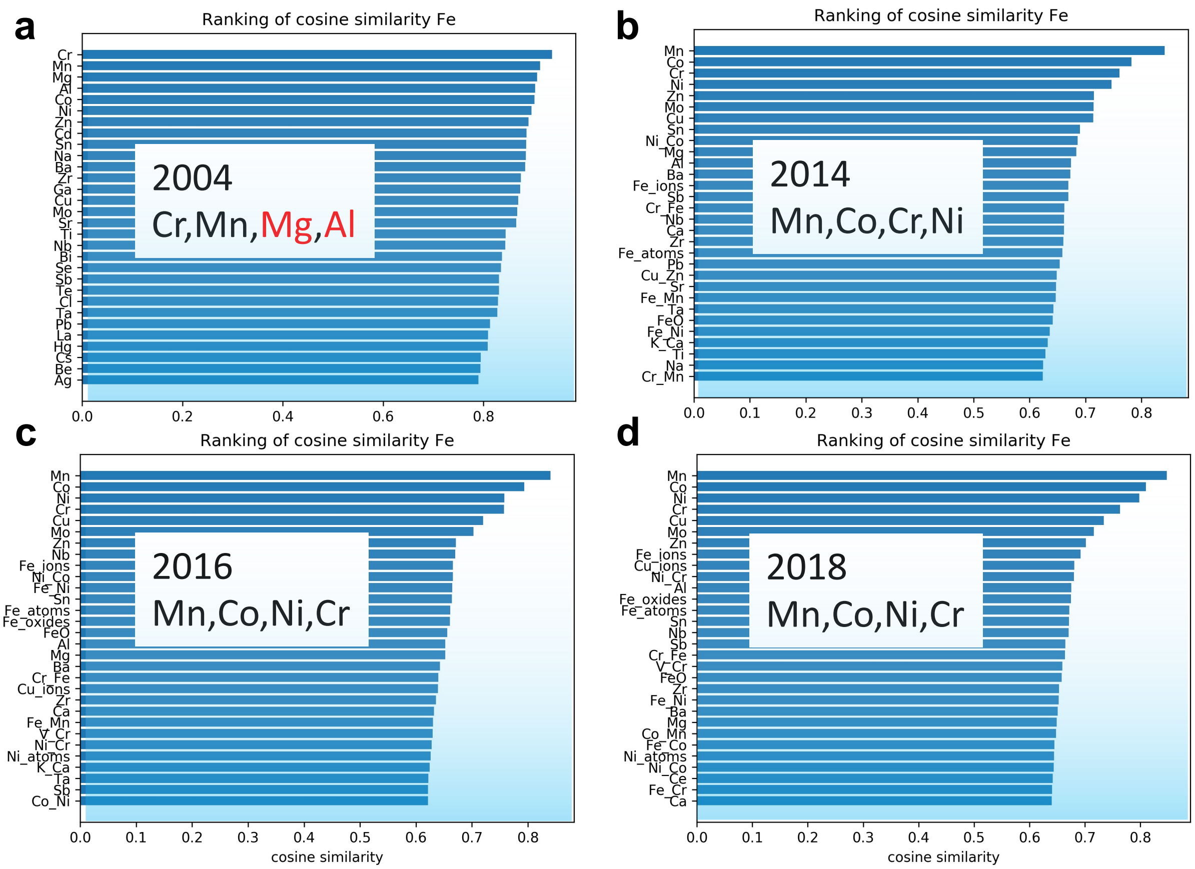

Supplementary Figure 4: Evolution of the most similar words for Fe. **a**, the year of 2004. **b**, the year of 2014. **c**, the year of 2016. **d**, the year of 2018.

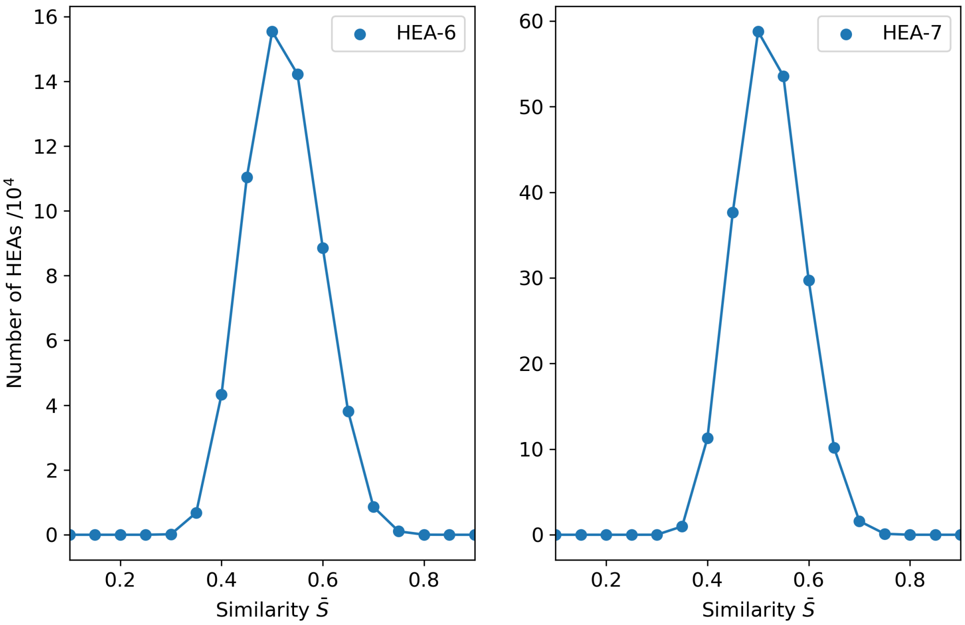

Supplementary Figure 5: Distribution of the similarity $\bar{S}$. Focusing on the 30 transition metal elements, we calculate the $\bar{S}$ for all alloy candidates of the six-component HEAs (left) and seven-component HEAs (right).

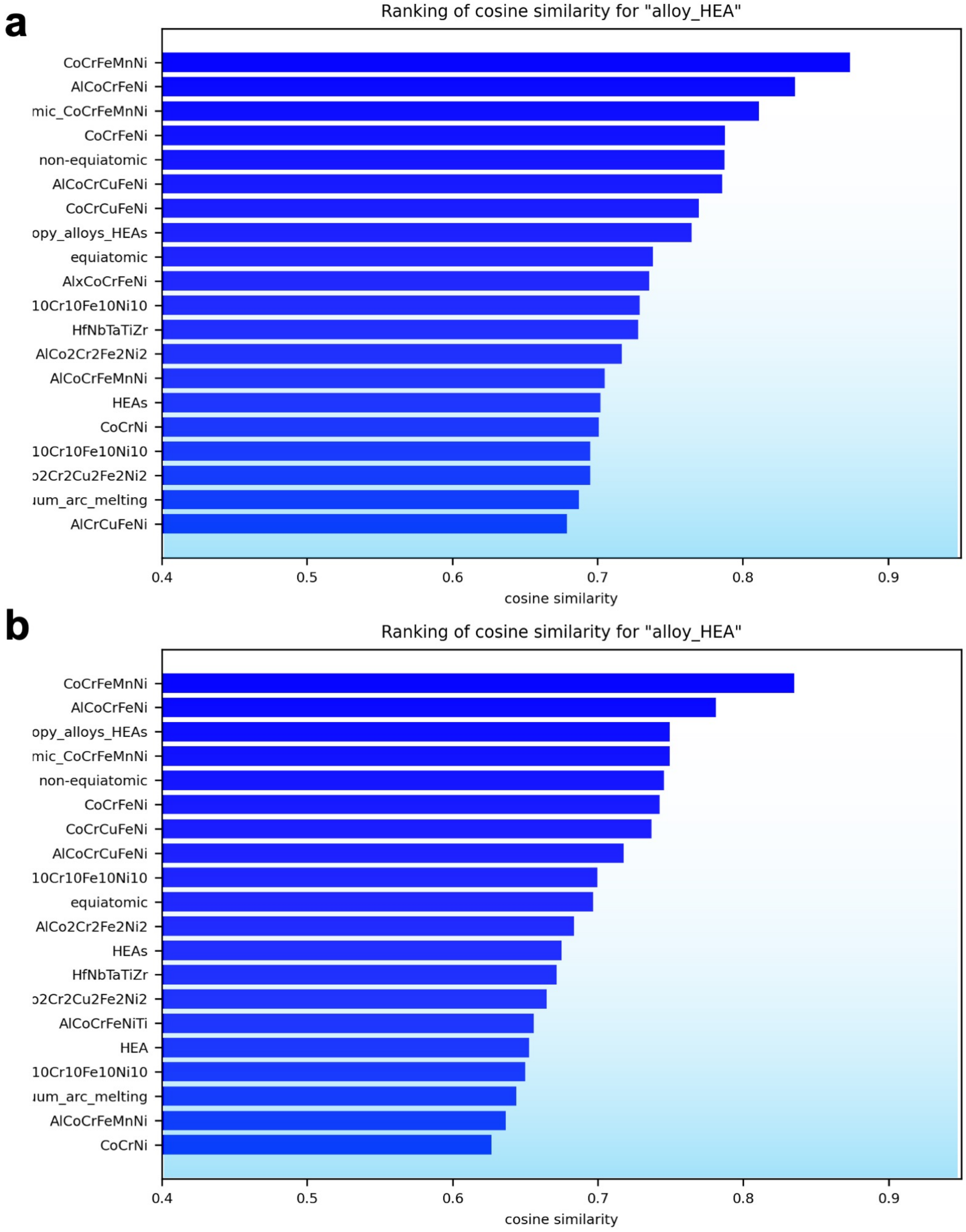

Supplementary Figure 6: Comparison of the most similar words using different skip-gram models. One model takes 200 for its dimension of word vectors and 8 for its widow size in the skip-gram algorithm (**a**). In contrast, the two parameters take values 300 and 10 for the second model (**b**). Taking the word ”alloy_HEA” as an example, we find its 20 most similar words. Most of these words identified by both models are the same and follow a similar order, such as CoCrFeMnNi, AlCoCrFeNi, CoCrFeNi, HEA, etc. This trend demonstrates a model with 200-dimensional word vectors and a window size of 8 is reliable for representing the existing knowledge in texts.

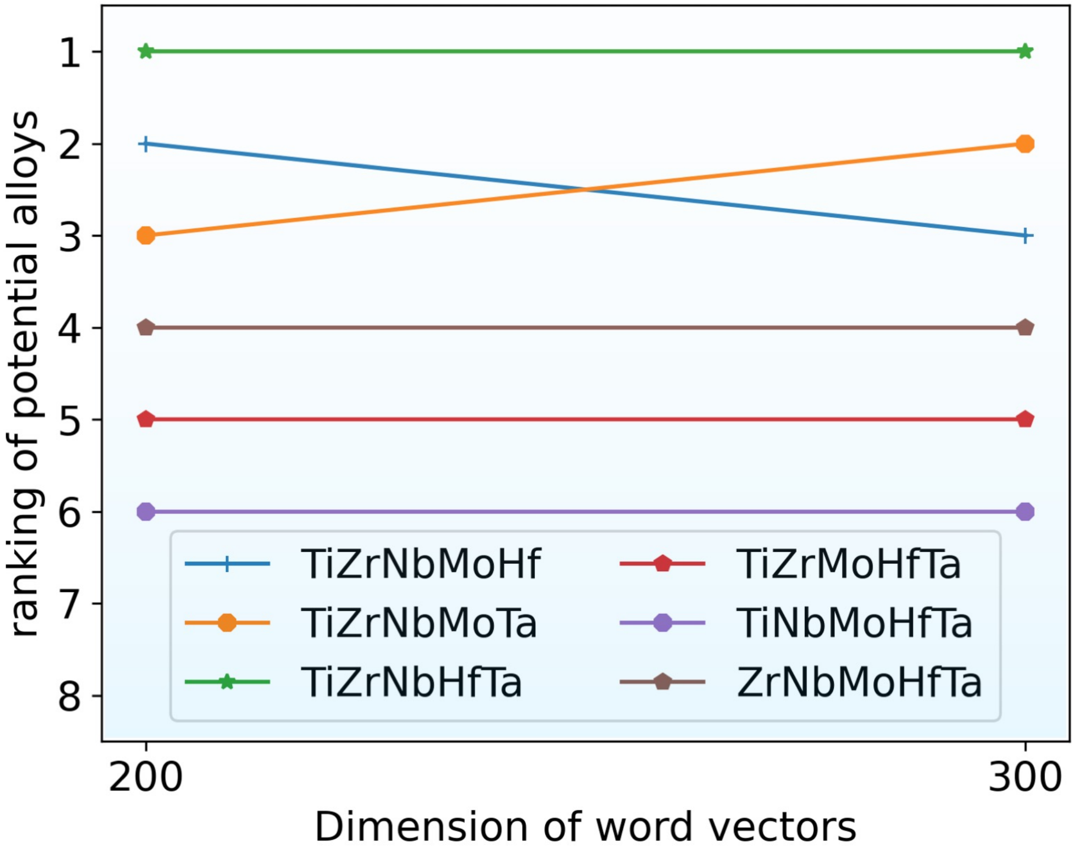

Supplementary Figure 7: Comparison of the alloy ranking using different skip-gram models. One model takes 200 for its dimension of word vectors and 8 for its widow size in the skip-gram algorithm. In contrast, the two parameters take values 300 and 10 for the second model. Taking BCC HEAs as examples, we calculate the similarity $\bar{S}$ and rank them according to this value. In both models, the representative Senkov alloy TiZrNbHfTa is ranked number one. All these HEAs follow the same order except for the TiZrNbMoHf and TiZrNbMoTa to switch their positions. This trend shows a model with 200-dimensional word vectors and a window size of 8 is sufficient for designing new alloys.

Supplementary Table 1

Supplementary Table 1: The predicted yield stresses for the FCC HEAs at 0K, 300K and 600K. The first seven columns are the alloy concentrations in atomic percent. The last three columns are the yield stresses in MPa.

V	Cr	Mn	Fe	Co	Ni	Cu	τ_y (0K)	τ_y (300K)	τ_y (600K)
20.0	20.0	20.0	20.0	20.0	0.0	0.0	650.0	493.0	374.0
20.0	20.0	20.0	20.0	0.0	20.0	0.0	595.0	447.0	336.0
20.0	20.0	20.0	20.0	0.0	0.0	20.0	667.0	522.0	408.0
20.0	20.0	20.0	0.0	20.0	20.0	0.0	581.0	435.0	325.0
20.0	20.0	20.0	0.0	20.0	0.0	20.0	651.0	507.0	395.0
20.0	20.0	20.0	0.0	0.0	20.0	20.0	560.0	429.0	329.0
20.0	20.0	0.0	20.0	20.0	20.0	0.0	657.0	499.0	379.0
20.0	20.0	0.0	20.0	20.0	0.0	20.0	751.0	594.0	470.0
20.0	20.0	0.0	20.0	0.0	20.0	20.0	666.0	521.0	407.0
20.0	20.0	0.0	0.0	20.0	20.0	20.0	649.0	505.0	393.0
20.0	0.0	20.0	20.0	20.0	20.0	0.0	627.0	465.0	344.0
20.0	0.0	20.0	20.0	20.0	0.0	20.0	755.0	590.0	461.0
20.0	0.0	20.0	20.0	0.0	20.0	20.0	690.0	534.0	413.0
20.0	0.0	20.0	0.0	20.0	20.0	20.0	675.0	520.0	401.0
20.0	0.0	0.0	20.0	20.0	20.0	20.0	765.0	598.0	468.0
0.0	20.0	20.0	20.0	20.0	20.0	0.0	232.0	146.0	91.0
0.0	20.0	20.0	20.0	20.0	0.0	20.0	394.0	284.0	205.0
0.0	20.0	20.0	20.0	0.0	20.0	20.0	339.0	239.0	168.0
0.0	20.0	20.0	0.0	20.0	20.0	20.0	324.0	226.0	158.0
0.0	20.0	0.0	20.0	20.0	20.0	20.0	398.0	287.0	207.0
0.0	0.0	20.0	20.0	20.0	20.0	20.0	320.0	217.0	148.0

Supplementary References

[1] Varvenne, C., Luque, A. & Curtin, W. A. Theory of strengthening in fcc high entropy alloys. *Acta Materialia* **118**, 164 – 176 (2016). URL http://www.sciencedirect.com/science/article/pii/S1359645416305481.

[2] Cantor, B., Chang, I., Knight, P. & Vincent, A. Microstructural development in equiatomic multicomponent alloys. *Materials Science and Engineering: A* **375**, 213–218 (2004).

[3] Chen, H. *et al.* Effect of the substitution of co by mn in al-cr-cu-fe-co-ni high entropy alloys. In *Annales De Chimie-Science Des Materiaux*, vol. 31, 685–698 (Paris; New York: Masson, 1978-, 2006).

[4] Zhang, Y., Zhou, Y. J., Lin, J. P., Chen, G. L. & Liaw, P. K. Solid-solution phase formation rules for multi-component alloys. *Advanced engineering materials* **10**, 534–538 (2008).
