# Supplementary material for: Toward the design of ultrahigh-entropy alloys via mining six million texts
Source: Nat Commun. 2023 Jan 4;14:54. doi: 10.1038/s41467-022-35766-5 (PMC9813346; doi:10.1038/s41467-022-35766-5)
Supplement: Supplementary file 2 — Description of Supplementary Data 1 [file 41467_2022_35766_MOESM2_ESM.docx]

File Name: Supplementary Data 1

Description: The file “selected-HEAs-equiatomic-lightweight.xlsx” includes the 494 lightweight ultrahigh-entropy alloys designed by the method proposed in this study.
